# Therapeutic development to accelerate malaria control through intentional intervention layering

**DOI:** 10.1186/s12936-024-05222-4

**Published:** 2025-01-13

**Authors:** Lydia Braunack-Mayer, Narimane Nekkab, Josephine Malinga, Sherrie L. Kelly, Evelyn Ansah, Joerg J. Moehrle, Melissa A. Penny

**Affiliations:** 1 Swiss Tropical and Public Health Institute, Allschwil, Switzerland; 2 University of Basel, Basel, Switzerland; 3 The Kids Research Institute Australia, Nedlands, WA Australia; 4 Centre for Child Health Research, University of Western Australia, Crawley, WA Australia; 5 University of Health and Allied Sciences, Ho, Ghana; 6 Medicines for Malaria Venture, Geneva, Switzerland

**Keywords:** *Plasmodium falciparum*, Malaria, Intervention layering, Intervention mixing, Modelling

## Abstract

The clinical development of novel vaccines, injectable therapeutics, and oral chemoprevention drugs has the potential to deliver significant advancements in the prevention of *Plasmodium falciparum* malaria. These innovations could support regions in accelerating malaria control, transforming existing intervention packages by supplementing interventions with imperfect effectiveness or offering an entirely new tool. However, to layer new medical tools as part of an existing programme, malaria researchers must come to an agreement on the gaps that currently limit the effectiveness of medical interventions for moderate to low transmission settings. In this perspective, three crucial gaps that may prevent new therapeutics from being used to their fullest extent are presented. First, do burden reduction outcomes, which are typically monitored in studies of new medical products, sufficiently capture the broader goal of accelerating malaria control? Layering novel malaria products requires monitoring health outcomes that reflect the novel product’s targeted stage of the parasite life cycle, in addition to all-infection and prevalence-based outcomes. Second, what public health outcome does a novel medical prevention tool provide that existing malaria interventions cannot fully deliver? Novel medical tools should be developed not just for an incremental improvement in preventive efficacy over an existing product, but also to meet a gap in protection. Specifically, this means designing products with components that target parts of the parasite life cycle beyond the scope of existing therapeutics, and better addressing populations and settings not well covered by existing tools. Finally, when do the population-level benefits of a multi-tool prevention programme justify the individual-level outcomes from receiving multiple interventions? An individual-level perspective should be key for exploring when and how layering a novel prevention intervention can accelerate efforts towards *P. falciparum* malaria control.

## Background

For many years, malaria control programmes have layered multiple prevention tools to protect populations from malaria. That is, simultaneously or sequentially implementing multiple interventions, creating a multifaceted defence system that targets various stages of the vector and parasite life cycle, and its associated burden. Recently, it has been postulated that layering malaria interventions could lead to a paradigm shift in malaria control. In the context of vector control, Paaijmans and Lobo argued that elimination efforts require a shift from a burden-focused response to addressing the “causative gaps in protection”, which are the systematic drivers of residual parasite transmission [1]. Ongoing research and development in vector control, such as gene drive techniques and *Wolbachia*-infected mosquitoes, could contribute new tools to control mosquito populations and reduce parasite transmission [2].

Different challenges should, however, be considered for achieving a paradigm shift in malaria control within the context of medical prevention. In other endemic infectious diseases, especially smallpox, elimination efforts have been facilitated by the availability of highly protective, long-lasting vaccines that provide sterile immunity [3]. For malaria, a similar therapeutic could potentially enable the scale-down of vector control and malaria chemoprevention. However, developing a vaccine with sterile immunity for malaria faces significant challenges, due to the complex life cycle and genetic diversity of *Plasmodium* parasites coupled with an incomplete understanding of the immune response. Since the development of such a product is not realistic in the near future, multiple, imperfect prevention therapeutics will need to be used in combination.

**Fig. 1:**
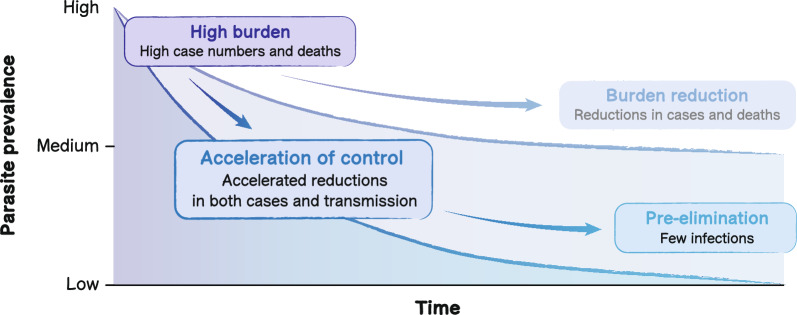
Malaria burden scenarios on the continuum to elimination

This perspective explores how new, imperfect therapeutics for preventing malaria can be best combined to accelerate malaria control. The term ‘acceleration of malaria control’ is used to refer to a scenario where a region accelerates reductions in parasite transmission and malaria morbidity through a set of interventions optimised for the region’s local transmission patterns, malaria burden, and health system capacities (Fig. 1). This perspective focuses on therapeutics that target *Plasmodium falciparum* malaria, as this parasite species continues to drive the majority of malaria burden [4]. Transformative tools that may replace a suite of existing tools are not specifically addressed, such as a transformative vaccine providing very long-term protection across all parasite life-cycle stages. Instead, this perspective explores why the intentional identification of gaps in the existing toolkit is critical to guide the development of new therapeutics for malaria control.

This perspective focuses on the use of multiple, imperfect prevention therapeutics in combination to lead to ‘acceleration of control’: accelerated reductions in parasite transmission and malaria morbidity through a set of interventions optimized for the region’s local transmission patterns, malaria burden, and health system capacities. Burden reduction refers to business as usual without optimised or intentionally layered intervention tools that fill gaps in the existing toolkit for malaria prevention.

## Main text

### Recent advances in medical research and development for *Plasmodium falciparum* malaria

**Fig. 2:**
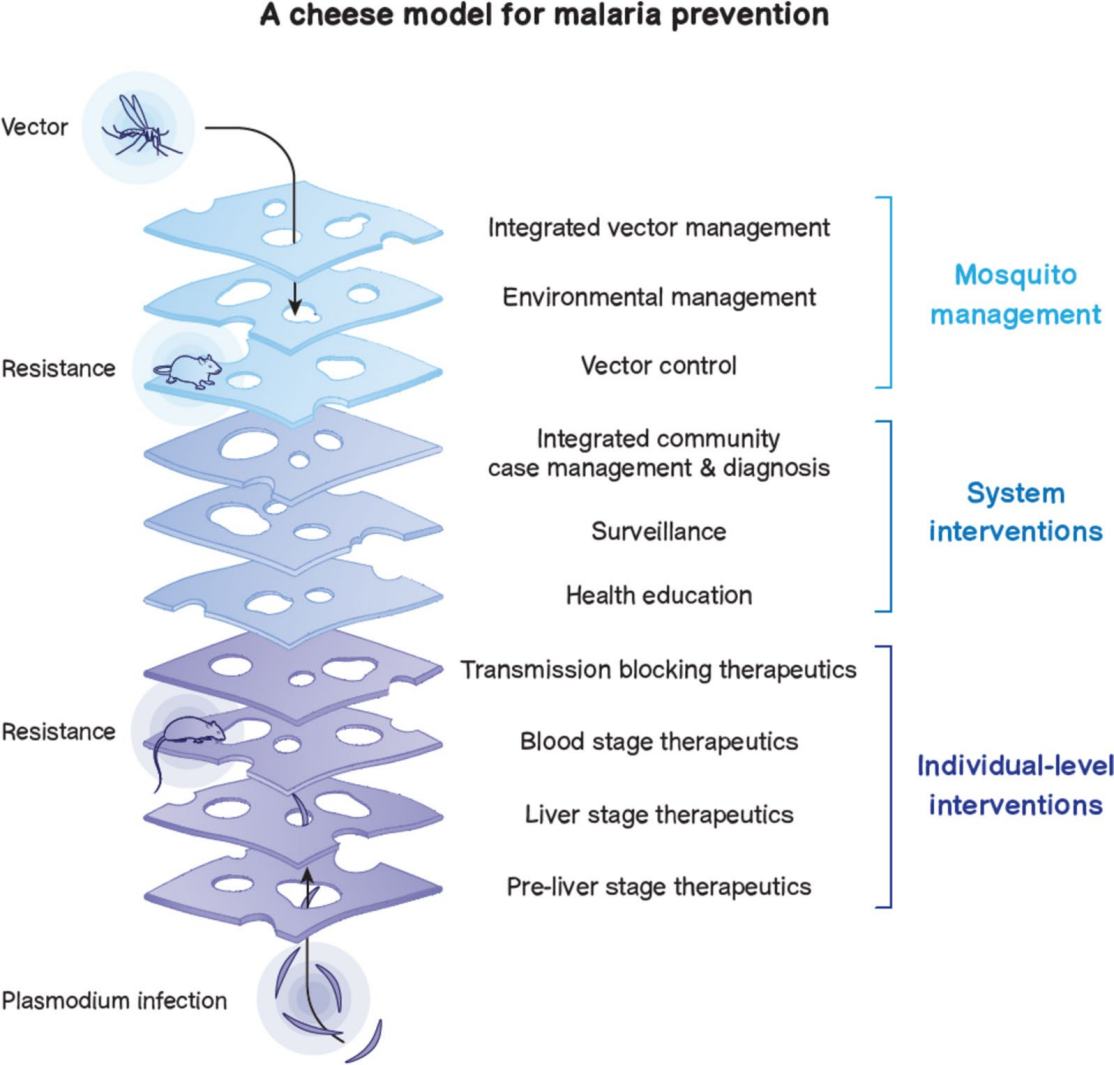
A cheese model for *Plasmodium falciparum* malaria prevention

The community’s toolkit for combating *P. falciparum* burden on the African continent currently layers long-lasting insecticidal nets (LLINs) and indoor residual spraying (IRS) for vector control, sulfadoxine-pyrimethamine (SP) with and without amodiaquine (AQ) for chemoprevention in children and pregnant women, and artemisinin-based combination therapy (ACT) with novel partner drugs, such as artesunate-pyronaridine, for treatment. Combining these vertical interventions with horizontal health system strategies, including integrated vector management and integrated community case management, enables an approach to malaria control where each layer complements the other components. This approach is illustrated in a ‘cheese model’ [5, 6] of malaria prevention (Fig. 2), where LLINs, chemoprevention, ACT, diagnostics, and IRS complement each other by tackling different parts of the parasite life cycle.

These interventions were supported by a renewal of global investments and attention towards malaria eradication following declarations at the Bill & Melinda Gates Foundation Malaria Forum in October 2007 [7], including substantial funding to purchase and deploy interventions [8–10]. Since 1999, product development partnerships have been established [11] to stimulate research and development and access activities for malaria drugs [12], vaccines [13], vector control [14], and diagnostics [15]. However, despite the significant investments made into supporting mass interventions and product development, the ongoing emergence of drug and insecticide resistance highlights the need for new therapeutics to achieve the ultimate goal of malaria eradication.

There are many different malaria interventions, each targeting a different part of the parasite life cycle from vector to parasite to clinical disease. The cheese model, adapted from a prevention model for SARS-CoV-2 [5, 6], recognises that each intervention is imperfect, whether it be due to parasite resistance, an intervention’s profile, or human behaviour, and no intervention is more important than any other. But, together, layering imperfect interventions can create a robust programme to accelerate towards malaria control.

This decade’s pharmaceutical development has promised both new products to replace imperfect drugs and new tools to reduce malaria burden and accelerate malaria control. The 2021 policy recommendation by the World Health Organization (WHO) for RTS,S/AS01_E_ [16] marked the first recommendation of a vaccine for malaria prevention, followed in 2023 by the recommendation for R21/Matrix-M [17]. While the approval of RTS,S prompted hope for a new tool to reduce malaria mortality in children, initial supply of this pre-liver stage vaccine was limited [18] and intervention costs are high relative to chemoprevention and bed net interventions [19]. Furthermore, since both RTS,S and R21 provide only partial protection against a single stage in the parasite life cycle, it is unlikely that malaria vaccination programmes will be able to fully replace other malaria prevention measures.

Beyond RTS,S and R21, attention has focused on advances in pre-liver stage monoclonal antibodies for malaria prevention [20, 21]. Other vaccine candidates [22], alternative monoclonal antibodies [23], and long-acting injectable and oral small molecule drugs [24, 25] are also being developed for use as malaria prevention tools. If deployed as part of a novel intervention, these new therapeutics may be able to complement the pre-liver stage malaria vaccines by providing liver stage, blood stage, or transmission blocking activity, thus offering protection against additional components of the within-host parasite life cycle. If used to replace the product used an in existing intervention, such as a SP-AQ for chemoprevention, novel therapeutics may be able to accelerate malaria control by contributing to substantial improvements in intervention delivery or access. But, in a landscape where both funding and product availability are limited, developers need to anticipate how malaria programs can best use new therapeutics to accelerate high burden regions towards malaria control.

### A new paradigm for acceleration of control

#### Are we assessing the right health outcomes to accelerate malaria control?

In countries with high malaria burden, prevention programmes necessarily focus on lowering childhood mortality and morbidity. Malaria programmes thus require therapeutics that prevent and treat clinical malaria cases and mortality. As a result, clinical trials and observational studies for malaria intervention layering have been designed to measure clinical cases and mortality in the populations most vulnerable to infection and disease. This has been the case even when the therapeutic being evaluated has a mechanism of action that does not target the parasite’s blood stage. For example, RTS,S targets the parasite’s pre-liver stage and acts by preventing sporozoites from causing liver-stage infection, which suggests a need for trials with infection endpoints. However, the primary outcome of the first randomized, controlled trial of seasonal RTS,S vaccination with seasonal malaria chemoprevention (SMC) was a malaria morbidity outcome [26, 27]. An ongoing trial of RTS,S combined with perennial malaria chemoprevention (PMC) will again measure morbidity efficacy endpoints in children [28].

In addition, a number of modelling studies have looked at how interventions can be layered to improve or optimize public health outcomes [29–32], also concentrating on achieving burden reduction goals in children through an optimised basket of interventions. Together with other malaria modelling studies, these studies have evaluated which combinations of interventions can achieve optimised outcomes with limited funding. It is important to acknowledge that questions around cost-effectiveness are critical to understand how layering interventions can accelerate a region towards malaria control, and that modelling may not be the best tool to explore these questions. However, when it comes to combining interventions for control acceleration, measures of cost-effectiveness that are only based on reductions in clinical cases and mortality may not be sufficient.

More specifically, using novel malaria interventions for control acceleration requires measuring a broader range of outcomes that are context-specific and tailored to the stage of the parasite life cycle that a product targets. Measuring burden reduction outcomes has meant that malaria policy decisions have been limited to cost-effectiveness evidence on a cost per clinical case or disability-adjusted life years (DALYs) averted, such as for novel insecticide-treated bed nets (ITNs) [33] and for the RTS,S [34] and R21 [35] vaccines. However, a broad policy recommendation on a cost per DALYs averted will not support an understanding of where novel therapeutics can be used to accelerate a region towards malaria control. As argued by Musoke and colleagues, outcome measures must be comprehensive enough to support “context specific” decisions, “considering how various attributes such as mosquito density and behaviour, existing evidence on proposed designs, and coverage of other malaria prevention approaches impact the mosquito or disease related outcomes” [36].

For example, Gavi bases its vaccine investment decisions for other endemic infectious diseases, such as polio, hepatitis B, and measles, on a comprehensive assessment of health indicators. Their assessment includes a vaccine’s impact on cases and deaths, its benefits for women and girls, and its impact on antimicrobial resistance [37]. Evaluating a wide range of outcomes has allowed Gavi to better support context-specific decisions in better controlling these endemic diseases. For novel preventive therapeutics, it may become critical to measure impact on all-infection and prevalence-based outcomes in addition to burden reduction outcomes, as provided in Challenger and colleague’s 2021 modelling study of the potential impact of a transmission-blocking vaccine [38].

Assessing all-infection and prevalence-based outcomes is inherently complex, requiring either expensive clinical trials or well-calibrated transmission models to translate between clinical and prevalence-based outcomes. This complexity is further compounded by the dual role that novel prevention therapeutics may play: reducing malaria burden in high-transmission settings and also aiding regions in their transition towards elimination. Therefore, it may not be a matter of choosing one over the other. Novel therapeutics may need to be evaluated for both their efficacy in preventing severe disease and mortality, as well as their impact on all-infection and prevalence-based outcomes. Hence, there is a need for agreement within the medical, malaria funding, policy, and research and development communities on how to prioritise clinical endpoints for new prevention therapeutics. This necessitates consensus among stakeholders with diverse incentives and goals. Without such consensus, product development investments may be wasted by overlooking interventions that are uniquely suited for different stages in the malaria control continuum, such as those targeting low-density or asymptomatic infections for ongoing transmission.

#### What public health outcome does a novel medical prevention tool provide that existing malaria interventions cannot fully deliver?

Integrating a novel intervention into an existing programme is a major undertaking. Ideally, effectiveness should be maximised for existing tools before time and resources are allocated to developing a new intervention layer. However, this has not always been the case.

The large-scale trial of seasonal vaccination with or without SMC indicates an intervention layering with a new tool where maximisation was not achieved. This trial evaluated the incidence of clinical malaria in children under five who received seasonal RTS,S delivery with or without SMC, at trial sites in Burkina Faso and Mali [26, 27]. At the five-year follow up, a protective efficacy of −3.0% (−11.8–5.2) was observed for RTS,S relative to SMC alone and 57.7% (53.3–61.7) for the combination relative to SMC alone. As Cairns and colleagues note in their secondary analysis, “the fact that SMC was not superior in the trial, despite high coverage of both interventions […] likely reflects a four-cycle SMC programme being used in areas where the epidemiology requires at least five [cycles] of SMC” [39]. In this example, prioritising SMC – an existing component of a malaria programme – may have reduced the number of malaria cases without the addition of RTS,S. Or, the combination of RTS,S and SMC could have been shown to be more beneficial had SMC been optimised first.

There are also access, delivery, and health-systems challenges to consider when developing a novel intervention to provide a benefit that existing interventions cannot fully deliver. For example, in Ghana, a dramatic increase in malaria vaccine coverage has been observed following the alignment of RTS,S’ fourth dose with the schedules of other childhood vaccines. The WHO’s recommendation for RTS,S specifies a three-dose primary schedule administered from five months of age, followed by a fourth dose 12 to 18 months later. The recommendation allows for flexibility in the schedule to optimise delivery [40], since the schedule specific for the fourth dose to be administered at either 22 or 24 months of age, outside of other immunization touchpoints [41]. Pilot countries Ghana, Malawi, and Kenya reported a high dropout rate between the third and fourth vaccine doses (30% in Ghana and Malawi, and 40% in Kenya) [42]. Ghana has since aligned the timing of the fourth dose with other childhood vaccines in the second year of life, achieving an increase in coverage of the fourth dose from 30 to 81% [42]. In this example, deploying the fourth dose of the new vaccine at its recommended schedule point may have led to reduced vaccine coverage, unintentionally reducing the potential health benefits of this new intervention.

**Fig. 3:**
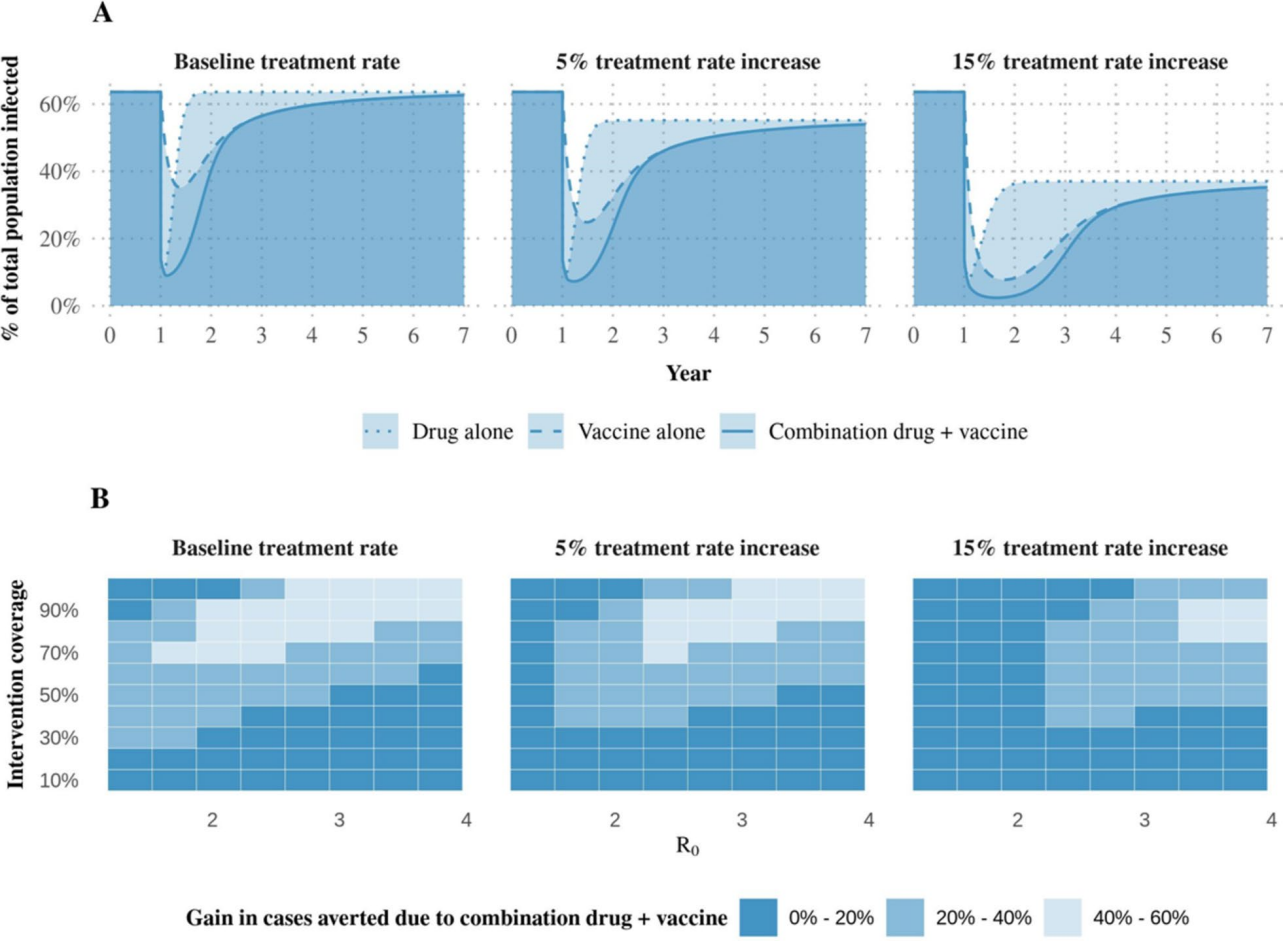
Results of a population-level disease model to illustrate the impact of layering malaria therapeutics. **A**: Illustrative impact of layering pulsed deployment of a chemoprevention drug and pre-liver stage vaccine on malaria prevalence. All interventions were deployed with 80% coverage, under constant transmission with an R_0_ of 2.75 (corresponding to all-ages annual prevalence of 64% under a no intervention counterfactual). The dotted line represents the change in malaria prevalence over time following drug administration, the dashed line represents change following vaccine administration, and the solid line represents change following administration of both interventions. Panels show varying rates of malaria treatment. **B**: Percentage point gain in cases averted during year one from layering a pre-liver stage vaccine on top of a pulsed chemoprevention drug. Gains in cases averted were calculated as ${(X}_{d} -{X}_{v}) / {X}_{n}$ for ${X}_{d}$ the total year one cases when a chemoprevention drug is deployed, ${X}_{v}$ the total year one cases occurring when both a drug and vaccine are pulsed to the full population, and ${X}_{n}$ the cases occurring if no intervention were deployed. These gains in percentage points are given for a range of coverages (y-axes) and levels of transmission (x-axes). The lighter the colour, the higher the additional impact achieved through layering interventions. For example, when a chemoprevention drug was given at 70% coverage in a setting with an R_0_ of 1.7, layering a vaccine with the same coverage led to a gain of more than 40% cases averted in the year following intervention

To illustrate the need to target novel therapeutics to address gaps in existing interventions, we used a simple theoretical, population-level disease model to explore the impact of layering malaria therapeutics on reductions in prevalence (Box 1). In Fig. 3, an SP-AQ-like drug was combined with an RTS,S-like pre-liver stage vaccine, deployed once en masse to the entire population (Table 1). While a completely hypothetical intervention package was modelled, results indicated that, when coverage of the mass drug deployment was imperfect and transmission was moderate or high, layering with a vaccine led to a substantial gain in cases averted. On the other hand, this gain was reduced in scenarios where the prevention gap left by the drug-based intervention was smaller: if drug coverage was improved, if transmission was reduced, or if treatment rates were increased. Results were consistent with an earlier modelling study [43], which found that synergies between layering mass deployment of a pre-liver stage vaccine and mass drug administration were lost when the latter intervention had high coverage.

**Table 1:** Intervention model parameterisations

Intervention	Effect	Coverage	Deployment
Chemoprevention drug	Based on SP-AQ’s protective efficacy profile, as in Burgert and colleagues, with a protective effect modelled with an initial efficacy of 100%, rapid decay, and a protective half-life of 31.1 days and a treatment effect modelled as clearing infection at the time of drug administration [48]	0% to 100%	Once at the start of year one
Pre-liver stage vaccine	Modelled with an initial efficacy of 91%, exponential decay, and a protective half-life of 7.3 months, or 222 days [34]	0% to 100%	Once at the start of year one
Improved treatment rates	Constant five or 15 percentage point increase in the recovery rate	100%	Continuously from the start of year one

**Table:** **Box 1 **A simple model for malaria dynamics.

We used a simple, population-level disease model to explore the impact of layering multiple malaria interventions. This simple model accounted for intervention deployment, decay, and transmission seasonality, but did not describe a number of key components of malaria dynamics: vector population dynamics, differences between uncomplicated and severe malaria, the acquisition of natural immunity, age, and the many other characteristics that make up real-world malaria dynamics. The simplicity of this model was, however, uniquely suited to serve as a proof-of-concept for malaria intervention layering, prompting questions that could be taken up with an individual-based model
Our model was based on the discrete form of the susceptible-infected-susceptible model, which is characterised by difference equations for transitions between malaria susceptible and infected states. Specifically, the number of infectious individuals at time $t$ was represented by
$${I}_{t+1}=\frac{\beta }{N}{I}_{t}(N-{I}_{t}) +\gamma {I}_{t}$$
for $\beta >0$ the number of successful contacts between infectious and susceptible individuals, $N$ the total population size, and $0<\gamma <1$ the proportion of the infectious population that remains infectious at each time step, and $t$ in days. We allowed the transmission parameter $\beta$ to represent the simplified, combined transmission of malaria in a human host to a mosquito vector and back again. The basic reproductive number ${R}_{0}$ without any intervention was given by $\frac{\beta }{1 - \gamma }$ and represented the number of secondary infections that resulted from a single infection
We extended this disease-agnostic model to include the effects of multiple malaria interventions: a chemoprevention drug, a pre-liver stage vaccine, and improved treatment rates. The number of infectious individuals at time was extended to
$${I}_{t+1}={\delta }_{t}\frac{\beta }{N}{I}_{t}(N-{I}_{t}) +{\alpha }_{t}(\gamma -{\varepsilon }_{t}){I}_{t}$$
for $0\le {\delta }_{t}\le 1$ the combined protective efficacy of the preventative interventions deployed and $0\le {\alpha }_{t},{\varepsilon }_{t}\le 1$ the impact of therapeutic administration and improved treatment, respectively, on the proportion of the population that remained infectious. Each intervention was deployed to all ages with the parameterization described in Table 1. We assumed a constant population of $10 000$ individuals and a baseline $\gamma$ of $0.984$, corresponding to $20\%$ access to treatment over two weeks. The impact of ongoing environmental management and vector control were represented by simulating a range of transmission levels for $\beta$ both constant and seasonally forced

There are a number of additional known gaps in malaria prevention interventions at the population level that new therapeutics could meet. These include gaps that existing interventions could potentially be extended to cover.

1. Pregnant women, where there is limited evidence of safety and efficacy data for anti-malarial drugs [44].
2. School-aged children residing in settings with perennial parasite transmission, where PMC is only recommended for children until two years of age [40].
3. Children and adolescents who may be at increased risk for malaria after ageing beyond eligibility for a chemoprevention or vaccination interventions [45].
4. Populations contributing to onwards transmission, including children and adults with asymptomatic infections.
5. Populations residing in previously low-risk settings with emerging mosquito prevalence, including urban and man-made environments where *Anopheles stephensi* is contributing to transmission [46] and regions where mosquito habitats are expanding due to climate change.

To accelerate malaria control, the funding, policy, and research and development communities need to agree on a set of such use cases for novel therapeutic tools. However, to the best of our knowledge, few novel tools are currently being developed with the specific goal of addressing one or more of these known gaps.

#### How does layering novel prevention tools benefit an individual, and when is this benefit worth the additional implications for individuals?

As discussed above, clinical, observational, and modelling studies have tended to quantify the benefits of intervention layering in terms of population-level outcomes, such as a reduction in symptomatic malaria cases. However, as the number of interventions deployed in a given population increases, so too does the potential for increased risk of safety concerns. The burden placed on the individual who receives multiple therapeutics also increases, as they invest time, effort, and resources into taking up multiple interventions. As such, there is a greater need to consider a caregiver and individual-level perspective when exploring when and how a novel prevention intervention can best accelerate malaria control.

Deploying a novel intervention on a population level will necessarily include some individuals who may not receive a direct or instantaneous benefit. For example, an adult receiving a malaria vaccine that only acts to block onwards transmission receives no direct benefit. A child with very high compliance to both nets and chemoprevention may also not directly benefit from receiving a malaria vaccine. Layered interventions that do not directly benefit the recipient will need to meet a high bar for safety and tolerability to minimise individual risk and thus increase acceptability and compliance.

Offering a therapeutic with a clear benefit to its recipient will also support intervention uptake when therapeutics are introduced in settings with other medical interventions. This is particularly critical for childhood interventions where high levels of coverage are required. For example, it was observed that children have missed doses of RTS,S as there are perceptions from caregivers that the number of childhood administrations is already too burdensome [47]. It is key to consider the preferences of target populations; individuals might prefer to receive simultaneous instead of separate interventions. It is the responsibility of product developers and regulatory agencies to ensure that the voices of individuals living in malaria endemic regions are heard throughout clinical development.

## Conclusions

In recent years, remarkable progress in pharmaceutical development has promised to accelerate malaria control efforts. While efforts have primarily concentrated on reducing symptomatic cases and mortality in high-burden areas, central questions remain regarding where and how to prioritise the integration of novel interventions into existing malaria programmes. Questions also remain as to when a novel therapeutic can replace a single or set of existing interventions. To navigate the complexities of layering with a novel malaria preventive therapeutic, the malaria research, development, and implementation community needs to agree on when layering novel interventions offers advantages that cannot be achieved through existing programmes. The individual's perspective must also be promoted, determining when the benefits of receiving a novel intervention outweigh the outcomes of receiving multiple therapeutics. And focus must be shifted from burden reduction alone to health outcomes that are close to the stage of the parasite life cycle that an intervention targets. Only by identifying and addressing these gaps, can novel interventions be layered for a paradigm shift from malaria burden to malaria control.

## Data Availability

The code and datasets supporting the conclusions of this article are available from GitHub via https://github.com/lydiab-mayer/malaria-intervention-layering/releases/tag/v1.0.0.

## Abbreviations

LLIN: Long-lasting insecticidal net
IRS: Indoor residual spraying
SP: Sulfadoxine-pyrimethamine
AQ: Amodiaquine
ACT: Artemisinin-based combination therapy
SMC: Seasonal malaria chemoprevention
PMC: Perennial malaria chemoprevention
ITN: Insecticide-treated bed net
